# Efficacy and safety of dronedarone in patients with a prior ablation for atrial fibrillation/flutter: Insights from the ATHENA study

**DOI:** 10.1002/clc.23309

**Published:** 2019-12-24

**Authors:** Mate Vamos, Hugh Calkins, Peter R. Kowey, Christian T. Torp‐Pedersen, Valérie Corp dit Genti, Mattias Wieloch, Andrew Koren, Stefan H. Hohnloser

**Affiliations:** ^1^ J.W. Goethe University, Department of Cardiology, Division of Clinical Electrophysiology Frankfurt Germany; ^2^ University of Szeged, Second Department of Medicine and Cardiology Center Szeged Hungary; ^3^ Johns Hopkins University Department of Medicine, Cardiology Baltimore Maryland; ^4^ Lankenau Heart Institute, Department of Cardiology Wynnewood Pennsylvania; ^5^ Nordsjaellands Hospital Department of Cardiology and Epidemiology Hillerød Denmark; ^6^ Sanofi‐Aventis Paris France; ^7^ Lund University Department of Clinical Sciences Malmö Sweden; ^8^ Sanofi US Inc. Bridgewater New Jersey

**Keywords:** ablation, antiarrhythmic drug, atrial fibrillation, atrial flutter, dronedarone

## Abstract

**Background:**

The role of antiarrhythmic drugs for atrial fibrillation/atrial flutter (AF/AFL) after catheter ablation is not well established.

**Hypothesis:**

We hypothesized that changing the myocardial substrate by ablation may alter the responsiveness to dronedarone.

**Methods:**

We assessed the efficacy and safety of dronedarone in the treatment of paroxysmal/persistent atrial fibrillation/atrial flutter (AF/AFL) post‐ablation, based on a post hoc analysis of the ATHENA study. A total of 196 patients (dronedarone 90, placebo 106) had an ablation for AF/AFL before study entry. In these patients, the effect of treatment on the first hospitalization because of cardiovascular (CV) events/all‐cause death was assessed, as was AF/AFL recurrence in individuals with sinus rhythm at baseline. The safety of dronedarone vs placebo was also determined.

**Results:**

In patients with prior ablation, dronedarone reduced the risk of AF/AFL recurrence (hazard ratio [HR]: 0.65 [95% confidence interval [CI]: 0.42, 1.00]; *P* < .05) as well as the median time to first AF/AFL recurrence (561 vs 180 days) compared with placebo. The HR for first CV hospitalization/all‐cause death with dronedarone vs placebo was 0.98 (95% CI: 0.62, 1.53; *P* = .91). Rates of treatment‐emergent adverse events were 83.1% vs 75.5% and rates of serious TEAEs were 27.0% vs 18.9% in the dronedarone and placebo groups, respectively. One death occurred with dronedarone (not treatment‐emergent) and five occurred with placebo.

**Conclusion:**

In patients with prior ablation for AF/AFL, dronedarone reduced the risk of AF/AFL recurrence compared with placebo, but not the risk of first CV hospitalization/all‐cause death. Safety outcomes were consistent with those of the overall ATHENA study.

**ABBREVIATIONS**
AAD: antiarrhythmic drug
ACE: angiotensin‐converting enzyme
AE: adverse event
AF: atrial fibrillation
AFL: atrial flutter
CA: cardiovascular
CI: confidence interval
HR: hazard ratio
ITT: intention‐to‐treat
LVEF: left ventricular ejection fraction
NYHA: New York Heart Association
SD: SD
TEAE: treatment‐emergent adverse event

## 1 INTRODUCTION

Antiarrhythmic drugs (AADs) have commonly been used as first‐line treatment for maintenance of sinus rhythm in individuals with paroxysmal and persistent atrial fibrillation (AF), and form a class IA recommendation in the American Heart Association (AHA)/American College of Cardiology (ACC)/Heart Rhythm Society (HRS) AF guidelines.^1^, ^2^ Real‐world data indicate an increasing use of catheter ablation for rhythm management of AF,^3^, ^4^ driven by improvements in ablation techniques and outcomes.^5^ In addition, recently completed clinical studies (including “Catheter Ablation vs Standard Conventional Treatment in Patients With LV Dysfunction and AF” [CASTLE‐AF; NCT00643188] and “Catheter Ablation vs Antiarrhythmic Drug Therapy in Atrial Fibrillation” [CABANA; NCT00911508]), comparing outcomes with first‐line catheter ablation vs medical therapy, support the safety of catheter ablation and highlight the potential for earlier ablation in selected patients.^6^, ^7^

The use of AADs and catheter ablation are not mutually exclusive.^1^, ^2^, ^5^ AADs are often used immediately following ablation to reduce the risk of early AF recurrence,^8^, ^9^, ^10^ and may be used in the long term depending on patient and physician preference.^5^ Successful ablation may alter the responsiveness to AADs,^11^ possibly as a result of changes to the substrate within the atrium and the triggers for AF. However, there is a substantial gap in scientific evidence regarding the impact of ablation on the effectiveness and safety of AADs in the post‐ablation setting.

Dronedarone is an AAD that has been shown to reduce the rate of hospitalization because of cardiovascular (CV) events in patients with paroxysmal or persistent AF or atrial flutter (AFL) in a randomized, double‐blind, placebo‐controlled phase 3 study (“A Trial with Dronedarone to Prevent Hospitalization or Death in Patients with Atrial Fibrillation” [ATHENA; NCT00174785]).^12^ The aim of the current analysis was to assess the efficacy and safety of dronedarone among patients with prior ablation who received treatment in the ATHENA study.

## 2 METHODS

### 2.1 Overview of the ATHENA study

ATHENA was a randomized, double‐blind, placebo‐controlled study conducted in 4628 patients in 551 centers across 37 countries. Details of the study design have been described previously.^12^, ^13^ Initially, patients were eligible for inclusion if they were at least 70 years of age or had a specified CV risk factor. In order to enrich the risk profile of patients, inclusion criteria were amended during the study to allow only patients ≥75 years old or ≥70 years old with at least one additional CV risk factor. Patients who experienced AF/AFL <6 months before randomization (based on 12‐lead electrocardiogram [ECG]) could be included if they had a second ECG showing sinus rhythm during this period. Patients could be enrolled while in sinus rhythm if conversion had occurred either spontaneously or after electrical or pharmacologic cardioversion. Patients enrolled while in AF/AFL were expected to undergo cardioversion after appropriate anticoagulation treatment.

Patients were randomized in a 1:1 ratio to dronedarone 400 mg twice daily or placebo in addition to their rate‐control therapy; the minimum follow‐up period was 12 months. The primary efficacy endpoint was first hospitalization because of CV events or death from any cause. Secondary endpoints included death from any cause, CV death and first CV hospitalization. The study was approved by the independent review board at each participating site and was conducted according to the Declaration of Helsinki. All patients provided written informed consent before participating in the study. Patient enrollment started in June 2005 and was completed in December 2006.

### 2.2 Post hoc analysis

This analysis focused on patients who had received any ablation procedure for AF/AFL before randomization in the ATHENA study and subsequently received study treatment. The primary and secondary endpoints of the ATHENA study were assessed in this patient population. First recurrence of AF/AFL was assessed in patients who were in sinus rhythm at baseline. In addition, the effect of ablation status on the incidence of treatment‐emergent adverse events (TEAEs) occurring from first study drug intake up to last study drug intake plus 10 days was assessed.

### 2.3 Assessments

Clinical evaluations, categorization for unplanned hospitalizations (CV or non‐CV), deaths (non‐arrhythmic cardiac, arrhythmic cardiac, non‐cardiac vascular, or non‐CV) and safety assessments were conducted as described previously.^12^

Scheduled 12‐lead ECGs were recorded at 7 and 14 days, and at 1, 3, and 6 months after randomization, then every 6 months thereafter. In addition, ECGs were recorded during unscheduled visits, such as in patients presenting with recurrent symptoms. Each ECG was classified by the investigator as demonstrating AF, AFL or sinus rhythm. AF/AFL recurrence was determined to occur at the first instance of cardioversion, AF/AFL based on electrocardiography or hospitalization for AF/AFL, as described previously.^14^

Patient baseline and demographic characteristics were reviewed for patients with and without ablation before study randomization.

### 2.4 Statistical analysis

For the primary endpoint, cumulative incidence functions in each treatment group were calculated and plotted using the Kaplan‐Meier method. Unadjusted hazard ratios (HRs) and 95% confidence intervals (CIs) were estimated using an unstratified Cox regression model with treatment group as the only factor. A multivariate analysis was considered unsuitable based on the small patient population and multiple biases associated with a post hoc analysis. Data were analyzed using SAS version 8.2 or later (Cary, North Carolina). Potential interaction between treatment (ie, dronedarone or placebo) and ablation status (ie, prior ablation or no prior ablation) was assessed using a Cox regression model for occurrence of first TEAE applied to the whole study safety population. An independent data and safety monitoring board provided regular reviews of study data.

## 3 RESULTS

### 3.1 Baseline and demographic characteristics

Among the 4628 patients randomized in the ATHENA study, 217 (4.7%) had received any ablation procedure before randomization, including 196 (4.2%) who had an ablation for AF/AFL. The proportion of patients who received ablation for AF/AFL before randomization was similar in the dronedarone (90 of 2301 patients; 3.9%) and placebo (106 of 2327 patients; 4.6%) groups (Table 1).

**Table 1:** Ablation status before randomization in the ATHENA study

Ablation status, n (%)	Dronedarone (n = 2301)	Placebo (n = 2327)	All (N = 4628)
No ablation	2203 (95.7%)	2208 (94.9%)	4411 (95.3%)
Ablation for AF/AFL	90 (3.9%)	106 (4.6%)	196 (4.2%)
Ablation only for AF/AFL	86 (3.7%)	102 (4.4%)	188 (4.1%)
Ablation for both AF/AFL and for other reason	4 (0.2%)	4 (0.2%)	8 (0.2%)
Ablation only for reason other than AF/AFL	8 (0.3%)	13 (0.6%)	21 (0.5%)

Abbreviations: AF, atrial fibrillation; AFL, atrial flutter.

Patient baseline and demographic characteristics in the dronedarone and placebo groups among patients with and without prior ablation are shown in Table 2. Among patients who had received prior ablation for AF/AFL, baseline and demographic characteristics were similar in the dronedarone and placebo groups.

**Table 2:** Patient baseline and demographic characteristics according to treatment group and prior ablation in the ATHENA study

	Patients with ablation for AF/AFL		Patients with no ablation	
	Dronedarone (n = 90)	Placebo (n = 106)	Dronedarone (n = 2203)	Placebo (n = 2208)
Age, mean years (SD)	70.1 (9.1)	68.2 (9.2)	71.6 (8.9)	71.8 (9.0)
Sex, male patients, n (%)	57 (63.3)	77 (72.6)	1109 (50.3)	1204 (54.5)
Race, n (%)				
White	77 (85.6)	96 (90.6)	1982 (90.0)	1965 (89.0)
Black	3 (3.3)	2 (1.9)	16 (0.7)	29 (1.3)
Asian	7 (7.8)	8 (7.5)	141 (6.4)	144 (6.5)
Other	3 (3.3)	0 (0)	64 (2.9)	70 (3.2)
Cardiovascular history, n (%)				
Hypertension	76 (84.4)	84 (79.2)	1915 (86.9)	1904 (86.2)
Structural heart disease	56 (62.2)	69 (65.1)	1267^a^ (58.0)	1324^b^ (60.6)
Tachycardia	46 (51.1)	56 (52.8)	703 (31.9)	731 (33.1)
Coronary heart disease	26 (28.9)	37 (34.9)	631 (28.6)	686 (31.1)
Non‐rheumatic valvular heart disease	20 (22.2)	20 (18.9)	311 (14.1)	330 (14.9)
Pacemaker	23 (25.6)	31 (29.2)	188 (8.5)	211 (9.6)
Lone AF	3 (3.3)	8 (7.5)	137^c^ (6.2)	130^c^ (5.9)
Ischemic dilated cardiomyopathy	6 (6.7)	11 (10.4)	85 (3.9)	106 (4.8)
Supraventricular tachycardia other than AF/AFL	8 (8.9)	7 (6.6)	84 (3.8)	79 (3.6)
Cardiac valve surgery	9 (10.0)	16 (15.1)	71 (3.2)	78 (3.5)
Non‐ischemic dilated cardiomyopathy	5 (5.6)	10 (9.4)	77 (3.5)	74 (3.4)
Hypertrophic cardiomyopathy	5 (5.6)	1 (0.9)	38 (1.7)	48 (2.2)
Implanted cardioverter defibrillator	4 (4.4)	11 (10.4)	37 (1.7)	31 (1.4)
Rheumatic valvular heart disease	2 (2.2)	5 (4.7)	49 (2.2)	24 (1.1)
Baseline cardiovascular examination, n (%)				
LVEF <35%	5^d^ (5.7)	7 (6.6)	87^e^ (4.0)	80^f^ (3.7)
Chronic heart failure symptoms (NYHA Class ≥1)	24 (26.7)	23 (21.7)	645 (29.3)	666 (30.2)
Baseline medications, n (%)				
Beta blockers (except sotalol)	66 (73.3)	72 (67.9)	1556 (70.6)	1560 (70.7)
ACE/angiotensin II inhibitor	56 (62.2)	72 (67.9)	1551 (70.4)	1522 (68.9)
Oral anticoagulant	69 (76.7)	81 (76.4)	1331 (60.4)	1293 (58.6)
Low‐dose aspirin	29 (32.2)	41 (38.7)	986 (44.8)	972 (44.0)
Statin	30 (33.3)	52 (49.1)	846 (38.4)	857 (38.8)
Calcium antagonist with heart‐rate‐lowering effect	17 (18.9)	18 (17.0)	314 (14.3)	286 (13.0)
Digitalis	17 (18.9)	18 (17.0)	304 (13.8)	287 (13.0)

*Note*: Denominators for calculation of percentages; ^a^n = 2184; ^b^n = 2185; ^c^n = 2199; ^d^n = 87; ^e^n = 2169; ^f^n = 2162.

Abbreviations: ACE, angiotensin‐converting enzyme; AF, atrial fibrillation; AFL, atrial flutter; LVEF, left ventricular ejection fraction; NYHA, New York Heart Association.

### 3.2 Efficacy

Median follow‐up for patients who received ablation for AF/AFL was 666 days in those treated with dronedarone and 688 days in those treated with placebo.

In patients with prior ablation, first CV hospitalization or death from any cause occurred in 35 of 90 patients (38.9%) randomized to dronedarone and in 42 of 106 patients (39.6%) randomized to placebo (HR: 0.98 [95% CI: 0.62, 1.53]; *P* = .91; Table 3). HR values for first CV hospitalization, death from any cause, and CV death were 1.05 (95% CI: 0.67, 1.66), 0.26 (95% CI: 0.03, 2.2) and 0.68 (95% CI: 0.06, 7.5), respectively (Table 3).

**Table 3:** Efficacy of dronedarone vs placebo in patients with ablation for AF/AFL before randomization in the ATHENA study

Event	No. of events/no. at risk		Median time to event, days (95% CI)			
	DRO	PBO	DRO	PBO	Hazard ratio (95% CI)	*P*‐value
First CV hospitalization or death from any cause	35/90	42/106	NR	NR	0.98 (0.62, 1.53)	.91
First CV hospitalization	35/90	39/106	NR	NR	1.05 (0.67, 1.66)	.83
Death from any cause	1/90	5/106	NR	NR	0.26 (0.03, 2.2)	.18
CV death	1/90	2/106	NR	NR	0.68 (0.06, 7.5)	.75
First AF/AFL recurrence^a^	36/63	46/65	561 (342, 778)	180 (61, 429)	0.65 (0.42, 1.00)	.048

Abbreviations: AF, atrial fibrillation; AFL, atrial flutter; CI, confidence interval; CV, cardiovascular; DRO, dronedarone; NR, not reached; PBO, placebo.

a Only patients in sinus rhythm at baseline included.

The most common cause of first CV hospitalization was AF/supraventricular rhythm disorders, accounting for 37% of first CV hospitalizations with dronedarone and 46% with placebo (Table S1). One death (because of cardiogenic shock) occurred in the dronedarone group and was not treatment‐emergent; five deaths (two sudden cardiac deaths, and one case each because of pneumonia, pancreatic adenocarcinoma, and multi‐organ failure) occurred in the placebo group.

**Figure 1:**
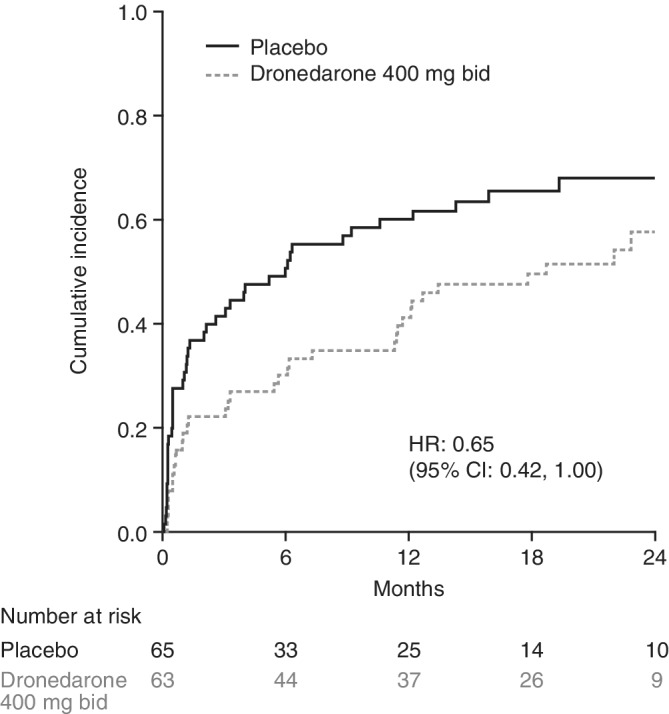
Cumulative incidence of first AF/AFL recurrence in patients with ablation for AF/AFL before randomization. (Only patients in sinus rhythm at baseline included). Abbreviations: AF, atrial fibrillation; AFL, atrial flutter; bid, twice daily; CI, confidence interval; HR, hazard ratio

Among the 128 patients who were in sinus rhythm at baseline, AF/AFL recurrence occurred in 36 of 63 patients (57.1%) treated with dronedarone and 46 of 65 patients (70.8%) treated with placebo (Table 3). Median time to first AF/AFL recurrence was longer (561 days [95% CI: 342, 778] vs 180 days [95% CI: 61, 429]) and risk of first AF/AFL recurrence was lower in patients treated with dronedarone compared with placebo (HR: 0.65 [95% CI: 0.42, 1.00], *P* < .05; Table 3; Figure 1).

### 3.3 Safety

TEAEs occurred in 83.1% and 75.5% of patients treated with dronedarone and placebo, respectively (Table 4). The most common TEAEs in the dronedarone and placebo groups were dizziness (12.4% vs 16.0%), diarrhea (14.6% vs 8.5%) and nausea (11.2% vs 3.8%). Serious TEAEs occurred in 27.0% of patients receiving dronedarone and in 18.9% of patients receiving placebo. Permanent study drug discontinuations because of adverse events (AEs) occurred in 10.1% of patients receiving dronedarone and 15.1% of patients receiving placebo. Using the Cox proportional hazard regression model on the whole study population, no evidence of interaction was shown between treatment (ie, dronedarone or placebo) and ablation status for occurrence of first TEAE (*P* = .44) or first serious TEAE (*P* = .14).

**Table 4:** Selected adverse events and laboratory abnormalities^a^

No. of patients, n (%)	Dronedarone (n = 89)	Placebo (n = 106)
Any TEAE	74 (83.1)	80 (75.5)
Cardiac events		
Any	10 (11.2)	14 (13.2)
Palpitations	1 (1.1)	6 (5.7)
Bradycardia	0 (0)	1 (0.9)
Respiratory events		
Cough	8 (9.0)	8 (7.5)
Dyspnea	9 (10.1)	6 (5.7)
Gastrointestinal events		
Diarrhea	13 (14.6)	9 (8.5)
Nausea	10 (11.2)	4 (3.8)
Endocrine events		
Hyperthyroidism	0 (0)	1 (0.9)
Neurologic events		
Dizziness	11 (12.4)	17 (16.0)
Headache	4 (4.5)	4 (3.8)
Skin‐related events		
Rash	2 (2.2)	2 (1.9)
Urticaria	1 (1.1)	0 (0)
Infections and infestations		
Upper respiratory tract infection	5 (5.6)	5 (4.7)
Urinary tract infection	5 (5.6)	1 (0.9)
Investigations		
Serum creatinine increase	1 (1.1)	2 (1.9)
QT prolongation	2 (2.2)	5 (4.7)
General disorders		
Peripheral edema	7 (7.9)	5 (4.7)
Asthenia	6 (6.7)	0 (0)
Chest pain	1 (1.1)	8 (7.5)
Any serious TEAE	24 (27.0)	20 (18.9)
Cardiac events	1 (1.1)	1 (0.9)
Respiratory events	3 (3.4)	1 (0.9)
Gastrointestinal events	5 (5.6)	5 (4.7)
Infections and infestations	4 (4.5)	4 (3.8)
Deaths^b^	0 (0)	2 (1.9)
AEs leading to permanent study drug discontinuation	9 (10.1)	16 (15.1)
QT prolongation	2 (2.2)	6 (5.7)^c^

Abbreviations: AE, adverse event; ITT, intention‐to‐treat; TEAE, treatment‐emergent adverse event.

a Time period from the first study drug intake up to the last study drug intake plus 10 days (“treatment period”); data include all AEs/laboratory abnormalities occurring in ≥5% of patients in either group plus selected AEs/laboratory abnormalities previously reported for the ITT ATHENA population.^12^

b Occurring from first study drug intake up to last study drug intake plus 10 days.

c One of these cases of QT prolongation leading to permanent study drug discontinuation occurred before first study drug intake.

## 4 DISCUSSION

In this analysis, dronedarone treatment decreased the risk of first AF/AFL recurrence compared with placebo in patients who had received catheter ablation before randomization, and demonstrated a safety profile similar to that observed in the overall ATHENA population.^12^ The rate of AF/AFL recurrence was 57% in the dronedarone group vs 71% in those treated with placebo.

The rates of first CV hospitalization or death from any cause (39% in the dronedarone group and 40% in the placebo group) in patients with prior ablation were in fact similar to those observed in the placebo arm of the overall ATHENA population (39%),^12^ and indicate a residual high burden of CV morbidity and mortality in these patients. For patients with prior ablation who were randomized to dronedarone, this outcome was driven predominantly by first CV hospitalization, with only one death from any cause. The most common causes of first CV hospitalization were AF/supraventricular rhythm disorders, which were numerically lower with dronedarone than with placebo.

Baseline and demographic characteristics of patients having prior ablation were broadly similar across the dronedarone and placebo groups. As expected, patients who had undergone ablation before randomization appeared more likely to be receiving an oral anticoagulant (77% vs 59%) and to have more extensive CV disease, including clinical tachycardia (52% vs 33%), a pacemaker implantation (28% vs 9%), cardiac valve surgery (13% vs 3%) or an implanted cardioverter defibrillator (8% vs 2%) at baseline than patients without ablation. Despite these differences, safety observations in patients with prior ablation were largely consistent with those in the overall ATHENA study.^12^ Relative risk analysis suggested no interaction between ablation status and treatment for either TEAEs or serious TEAEs.

With the increasing use of ablation to achieve rhythm control in patients with AF,^3^, ^4^ positioning of AADs and ablation in specific patient populations is of considerable practical importance. In current AHA/ACC/HRS guidelines, AADs remain a first‐line treatment recommendation for patients with paroxysmal and persistent AF, with choice of AAD depending on underlying heart disease and comorbidities.^1^, ^2^ Both AHA/ACC/HRS guidelines^1^, ^2^ and an expert consensus from HRS/EHRA/ECAS/APHRS/SOLEACE^5^ consider catheter ablation as a class IA recommendation in patients with symptomatic paroxysmal AF who are refractory or intolerant to at least one class I or III AAD, and as a IIaB recommendation as first‐line treatment in patients with paroxysmal AF, prior to initiation of an AAD.

In real‐world evaluations, fewer than 50% of patients achieve treatment success at 1 year following catheter ablation, based on freedom from AF recurrence without the use of AADs.^15^, ^16^ Data from the ESC‐EHRA AF ablation long‐term registry show that for patients receiving ablation, 90% had received AAD treatment before ablation, 68% received AADs shortly after ablation and 46% were on AADs at 1‐year follow‐up.^17^ In the CABANA study, a high incidence of crossovers between the ablation and medical therapy arms highlights the difficulty in separating ablation and medical therapies for AF rhythm management.^7^ Furthermore, almost 50% of patients in the ablation arm of the CABANA study had AF recurrences at 4 years, emphasizing the need to explore efficacy and safety of AAD treatment as complementary rather than competing approaches to rhythm management.

While short‐term adjunctive AAD therapy has been shown to significantly reduce the risk of early AF recurrence compared with ablation alone,^9^ there are few randomized controlled trials and real‐world data addressing outcomes with AADs in patients with prior ablation, and the efficacy and safety of AADs in this setting are unclear.^5^, ^18^, ^19^ Data from a large, single‐center study in 439 patients with paroxysmal or persistent AF suggest that shortened time to recurrence after ablation is linked to an increased responsiveness to AADs, possibly as a result of substrate changes, making reintroduction of AAD therapy after ablation a compelling option.^11^

### 4.1 Limitations

The ATHENA study was not designed or powered to detect differences in efficacy or safety of dronedarone vs placebo within the subset of patients with prior ablation. As a post hoc analysis, and with a relatively small number of patients, this study should be regarded as exploratory. Furthermore, information was not available on either the timing or characteristics of prior ablation, the type of AF/AFL for individual patients at randomization, or heart rate during sinus rhythm and AF episodes throughout the study.

## 5 CONCLUSION

In this post hoc analysis, dronedarone delayed the time to first AF/AFL recurrence in the subset of ATHENA patients with prior catheter ablation, with safety outcomes consistent with those of the overall ATHENA study. These data are hypothesis‐generating and support further evaluation of dronedarone in the post‐ablation setting in adequately sized prospective studies.

## CONFLICT OF INTEREST

Outside the submitted work, M.V. reports consulting fees and/or non‐financial support from Bayer, BMS, Daiichi Sankyo, Egis, Pfizer, Sanofi‐Aventis, SJM and Spectranetics; C.T‐P. reports consulting fees for Bayer; S.H.H reports consulting fees from Bayer, BI, BMS, Daiichi Sankyo, Sanofi‐Aventis, Pfizer and Zoll; and P.R.K. reports consulting fees from Sanofi‐Aventis. A.K., M.W. and V.d.G. are employees of Sanofi. H.C. has nothing to disclose.

## Supporting information


**Table S1** Reasons for first cardiovascular hospitalization in patients who received ablation for atrial fibrillation/atrial flutter (AF/AFL) before randomization in the ATHENA study
