# Supplementary material for: Efficacy and safety of dronedarone in patients with a prior ablation for atrial fibrillation/flutter: Insights from the ATHENA study
Source: Clin Cardiol. 2019 Dec 24;43(3):291–7. doi: 10.1002/clc.23309 (PMC7068068; doi:10.1002/clc.23309)
Supplement: Supplementary file 1 — Table S1 Reasons for first cardiovascular hospitalization in patients who received ablation for atrial fibrillation/atrial flutter (AF/AFL) before randomization in the ATHENA study [file CLC-43-291-s001.docx]

**Supplementary material**

**SUPPLEMENTARY TABLE 1** Reasons for first CV hospitalization in patients who received ablation for AF/AFL before randomization in the ATHENA study

|  | No. of patients with CV hospitalization (%) | | | |
| --- | --- | --- | --- | --- |
| **Reason for first CV hospitalization** | Dronedarone  (n = 90) | | Placebo  (n = 106) | |
| AF/other supraventricular rhythm disorders | 13 (14.4) | 18 (17.0) | |  |
| CV surgery except cardiac transplantation | 4 (4.4) | 3 (2.8) | |  |
| Worsening CHF, including pulmonary edema or dyspnea of cardiac origin | 4 (4.4) | 3 (2.8) | |  |
| Stable angina pectoris or atypical chest pain | 4 (4.4) | 2 (1.9) | |  |
| Syncope | 3 (3.3) | 0 | |  |
| Implantation of a pacemaker, ICD or any other cardiac device | 2 (2.2) | 3 (2.8) | |  |
| Transcutaneous coronary, cerebrovascular or peripheral procedure | 1 (1.1) | 6 (5.7) | |  |
| Major bleeding (requiring two or more units of blood or any intracranial hemorrhage) | 1 (1.1) | 1 (0.9) | |  |
| MI or unstable angina | 1 (1.1) | 1 (0.9) | |  |
| TIA or stroke (except intracranial hemorrhage) | 1 (1.1) | 1 (0.9) | |  |
| Blood pressure related (hypotension, hypertension; except syncope) | 1 (1.1) | 0 | |  |
| Ventricular tachycardia (non-sustained and sustained VT) | 0 | 1 (0.9) | |  |

Abbreviations: AF, atrial fibrillation; AFL, atrial flutter; CHF, congestive heart failure;
CV, cardiovascular; ICD, implantable cardioverter defibrillator; MI, myocardial infarction;
TIA, transient ischemic attack; VT, ventricular tachycardia.
